# Brain volume measured by synthetic magnetic resonance imaging in adult moyamoya disease correlates with cerebral blood flow and brain function

**DOI:** 10.1038/s41598-024-56210-2

**Published:** 2024-03-05

**Authors:** Kazufumi Kikuchi, Osamu Togao, Koji Yamashita, Takuro Isoda, Ataru Nishimura, Koichi Arimura, Akira Nakamizo, Koji Yoshimoto, Kousei Ishigami

**Affiliations:** 1 Department of Molecular Imaging and Diagnosis, Graduate School of Medical Sciences, Kyushu University, 3-1-1 Maidashi, Higashi-ku, Fukuoka, 812-8582 Japan; 2 Department of Clinical Radiology, Graduate School of Medical Sciences, Kyushu University, 3-1-1 Maidashi, Higashi-ku, Fukuoka, 812-8582 Japan; 3 Department of Neurosurgery, Graduate School of Medical Sciences, Kyushu University, 3-1-1 Maidashi, Higashi-ku, Fukuoka, 812-8582 Japan

**Keywords:** Image processing, Neurology

## Abstract

Moyamoya disease (MMD) is characterized by progressive arterial occlusion, causing chronic hemodynamic impairment, which can reduce brain volume. A novel quantitative technique, synthetic magnetic resonance imaging (SyMRI), can evaluate brain volume. This study aimed to investigate whether brain volume measured with SyMRI correlated with cerebral blood flow (CBF) and brain function in adult MMD. In this retrospective study, 18 adult patients with MMD were included. CBF was measured using iodine-123-N-isopropyl-p-iodoamphetamine single photon emission computed tomography. Cerebrovascular reactivity (CVR) to acetazolamide challenge was also evaluated. Brain function was measured using the Wechsler Adult Intelligence Scales (WAIS)-III/IV and the WAIS-R tests. Gray matter (GM), white matter, and myelin-correlated volumes were evaluated in six areas. Resting CBF was positively correlated with GM fractions in the right anterior cerebral arterial and right middle cerebral arterial (MCA) territories. CVR was positively correlated with GM fraction in the right posterior cerebral arterial (PCA) territory. Full-Scale Intelligence Quotient and Verbal Comprehension Index scores were marginally positively correlated with GM fractions in the left PCA territory. Processing Speed Index score was marginally positively correlated with GM fraction in the right MCA territory. The SyMRI-measured territorial GM fraction correlated with CBF and brain function in patients with MMD.

## Introduction

Moyamoya disease is a rare cerebrovascular disorder characterized by progressive occlusion of the internal carotid artery and its main branches within the circle of Willis^1^. This arterial occlusion causes chronic hemodynamic impairment, which can reduce brain volume and impair cognitive function and working performance due to myelin damage^2^. Previous studies have used magnetic resonance imaging (MRI) to evaluate the correlation between myelin loss and processing speed by comparing healthy individuals with patient groups^2–4^. However, conventional MRI cannot directly quantify myelin. This is because myelin contains few free water molecules and water protons in the myelin sheath, and water protons between the phospholipid bilayers have a short transverse relaxation time (TE, T2 ≃ 10 ms), making the signal acquisition with conventional MR scanner using long TE (≃ 50–100 ms) theoretically impossible^5^.

In contrast, a novel quantitative MR technique, synthetic MRI (SyMRI), can evaluate the brain volume, including myelin, in a clinically practical scan time^6^. SyMRI quantifies tissue relaxation properties to synthesize various types of images from a single acquisition^7^. The 3D-Quantification using an interleaved Look-Locker Acquisition Sequence (3D-QALAS) is a three-dimensional image type of SyMRI, which enables the simultaneous quantification of multiple relaxation times, such as T1 and T2, and quantifies the brain volumes, including gray matter (GM), white matter (WM), and myelin^6^. This new sequence is implemented in non-invasive evaluations of multiple sclerosis^8^; however, its application in moyamoya disease remains understudied. The existing literature on SyMRI predominantly discusses its accuracy and efficiency in quantifying myelin^9,10^, but its potential usefulness in moyamoya disease has not been widely explored. Furthermore, the interplay between regional brain volume changes and cerebral blood flow (CBF) has been extensively documented for various pathologies^11,12^, but not for moyamoya disease. We aimed to investigate whether SyMRI-measured brain volume correlated with CBF and brain function in adult patients with moyamoya disease, seeking to clarify their association with disease severity.

## Materials and methods

### Patients

This retrospective study was conducted in accordance with the principles embodied in the Declaration of Helsinki and was approved by the Institutional Review Board / Ethics Committee of Kyushu University (#2020-228). The requirement for the acquisition of informed consent from patients was waived by the Institutional Review Boards / Ethics Committees of Kyushu University owing to the retrospective nature of this study.

A total of 18 consecutive patients from 2019 to 2021 participated in this study. The inclusion criteria were as follows: (1) with bilateral moyamoya disease; (2) adults (age ≥ 18 years); (3) pre- or post-bypass operation; (4) with available MRI scans that included conventional imaging, T1WI, T2WI, FLAIR, and MRA, as well as SyMRI; and (5) with cerebral angiography and iodine-123-N-isopropyl-p-iodoamphetamine (^123^IMP) single-photon emission computed tomography (SPECT) data. The exclusion criterion was inappropriate image quality owing to severe artifacts or motion.

### Magnetic resonance imaging

MRI was performed using a 3-T MR scanner (Ingenia 3.0 T CX; Philips Healthcare, Best, Netherlands) with a 15-channel head coil. Quantitative MRI was performed using the 3D-QALAS sequence^6^. The imaging parameters were as follows: TR/TE, 4.9/2.2 ms; T2 prep echo time, 100 ms; inversion delay times, 100, 1000, 1900, and 2800 ms; FOV, 230 × 193 × 150 mm^3^, matrix, 192 × 161 (recon. 512 × 512); thickness, 1.2 mm; number of slices, 146; flip angle, 4º; bandwidth, 358 Hz/pixel; turbo field echo factor, 150; and total scan time, 6 min 6 s. Images obtained from the 3D-QALAS sequence were preprocessed on a prototype of the SyMRI software (version 0.45.34; SyntheticMR, Linköping, Sweden) to obtain GM, WM, and myelin-correlated (MyC) maps.

### Iodine-123-N-isopropyl-p-iodoamphetamine single photon emission computed tomography

The Iodine-123-N-isopropyl-p-iodoamphetamine single photon emission computed tomography (^123^IMP-SPECT) examination was performed using the dual-table autoradiographic (DTARG) method to calculate regional CBF (rCBF) sequentially before and after an acetazolamide challenge in a single session of SPECT, as previously described^13^. SPECT images were obtained with a double-head rotating gamma camera (Symbia T6: Siemens Medical Solutions USA, Inc., Hoffman Estates, IL, USA) with a parallel hole low-medium energy general purpose collimator. The spatial resolution was 10.6-mm full width at half maximum at 10 cm. The SPECT scans followed the DTARG protocol, with dual administration of ^123^I-IMP (Nihon Medi-Physics Co., Ltd., Nishinomiya, Japan). Two dynamic scans were acquired in quick succession, with a 2 min interval between the scans. The first scan covered the initial 0 to 28 min period, and the second was acquired from 30 to 58 min. At 4 min per frame, 7 frames covered each of the 2 dynamic scan periods. The ^123^I-iodoamphetamine (148 MBq) was infused twice over 1 min into the antecubital vein at 0 and 30 min. Acetazolamide (1000 mg) was administered intravenously at 20 min after the first IMP injection, corresponding to 10 min before the second IMP injection. Projection data were summed for the acquisition duration of the first and second scans and reconstructed using QSPECT software^14^. The matrix and pixel sizes of the SPECT images were 64 × 64 × 36 and 2.78 × 2.78 × 5.39 mm, respectively. The raw SPECT imaging data in the resting and acetazolamide challenge states for all patients were also acquired. To calculate rCBF after an acetazolamide challenge, two look-up tables were used to reflect the effect of radioactivity in the brain from the first dose of IMP. The data were transferred to the workstation, and the rCBF of the hemisphere was measured by creating a region of interest using 'NEURO FLEXER’ software (AZE Corporation, Kanagawa, Japan)^15^. The NEURO FLEXER volume-of-interest template contains 20 regions based on arterial territories, including the anterior cerebral arterial (ACA), middle cerebral arterial (MCA), and posterior cerebral arterial (PCA) territories. The rCBF and cerebrovascular reactivity (CVR) to an acetazolamide challenge were measured on SPECT. The CVR was measured based on the following equation:

$$ {\text{CVR}} = \left[ {{\text{CBF}}_{{{\text{ACZ}}}} {-}{\text{ CBF}}_{{{\text{rest}}}} } \right]/{\text{CBF}}_{{{\text{rest}}}} \times {1}00 \, \left( \% \right) $$

where CBF_ACZ_ is the CBF after the acetazolamide challenge and CBF_rest_ is the CBF at baseline. Using the rCBF measured by SPECT, the CBF ratio was calculated by the averaged CBF in the bilateral cerebellar hemispheres because it is not necessary to consider individual differences in drug uptake when using the CBF ratio on SPECT^15^.

### Brain functional test

Each patient was assessed with the Wechsler Adult Intelligence Scales (WAIS) III and IV and the Wechsler Adult Intelligence Scale-Revised (WAIS-R). The WAIS-III/IV has five parameters (1. Full-Scale Intelligence Quotient (FSIQ), 2. Verbal Comprehension Index (VCI), 3. Perceptual Reasoning Index (PRI), 4. Working Memory Index (WMI), and 5. Processing Speed Index (PSI)), and the WAIS-R also has five parameters (1. Verbal Memory Index, 2. Visual Memory Index, 3. General Memory Index, 4. Attention/Concentration, and 5. Delayed Recall). The neuropsychological tests were performed within seven days before SyMRI. All examinations were administered by a trained neuropsychologist who was blinded to the patient’s clinical information.

### Image analysis

**Figure 1:**
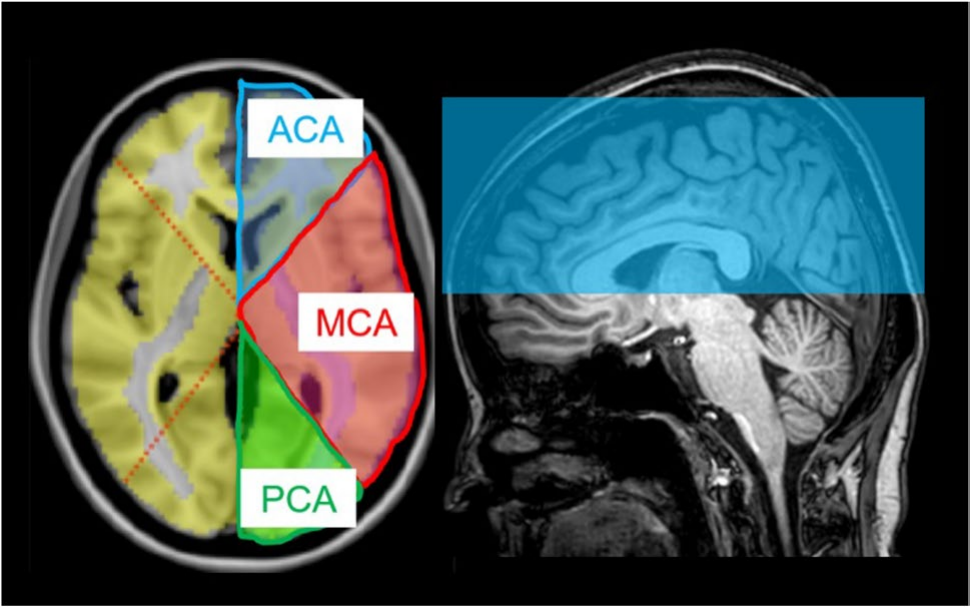
The regions of interest based on the Alberta Stroke Program Early CT Score (ASPECTS). The regions of interest are placed in the anterior cerebral arterial (ACA, blue), middle cerebral arterial (MCA, red), and posterior cerebral arterial (PCA, green) territories, roughly corresponding to the Alberta Stroke Program Early CT Score (ASPECTS) based on the Montreal Neurological Institute brain template. Each cerebral arterial territory is measured from the basal ganglia level to the vertex (right: blue area).

Volumetric analysis focused on three major arterial territories (ACA, MCA, and PCA), as reported in a previous study on moyamoya disease^2^. The ROIs were placed in the ACA, MCA, and PCA territories on each of the GM, WM, and MyC maps, roughly corresponding to the Alberta Stroke Program Early CT Score (ASPECTS) based on the Montreal Neurological Institute (MNI) brain template^16^. The ASPECTS system is a well-established method for evaluating early ischemic changes on CT^17,18^ and can evaluate even fine regions within vascular territories^19^. We first placed the center of the ROI on the interthalamic adhesion. Then, the same ROIs were used to measure the volume in all slices. We transformed the ASPECTS atlas into individual native space. Six ROIs were overlaid onto the bilateral hemispheres, and the brain volume from the basal ganglia to the vertex was measured and summed (Fig. 1). Each volume fraction (%) was calculated using the equation:

$$ {\text{Volume}}\;{\text{fraction}}\;\left( \% \right) = \,{\text{each}}\;{\text{volume}}\; \, \left( {{\text{GM}},\,{\text{WM}},\;{\text{MyC}}} \right){\text{/total}}\,{\text{ intracranial}}\;{\text{ volume}} $$

A total of 18 fractions were assessed (ACA/MCA/PCA × right/left hemisphere × GM/WM/MyC). We evaluated the correlations between brain volume fractions and brain blood flow and between brain volumes and brain function. In addition, we investigated the correlations of Suzuki’s stenosis grade with brain volume and CBF.

### Statistical analysis

Correlations between brain volume fraction and CBF/brain function were evaluated with Spearman's rank-order correlation. Statistical analyses were performed using Prism 7.0 (GraphPad Software, San Diego, CA). *P*-values < 0.05 were considered statistically significant. Bonferroni correction was performed for multiple tests to determine statistical significance according to the number of analyzed combinations (for 18 combinations, a Bonferroni correction coefficient of 18 was applied to the *P*-values).

### Ethics approval

All procedures performed in studies involving human participants were in accordance with the ethical standards of the institutional and/or national research committee and with the 1964 Helsinki Declaration and its later amendments or comparable ethical standards. This retrospective study was approved by our Institutional Review Board (Number 2020-228).

### Consent to participate

Consent to participate was waived due to the retrospective study design.

## Results

**Figure 2:**
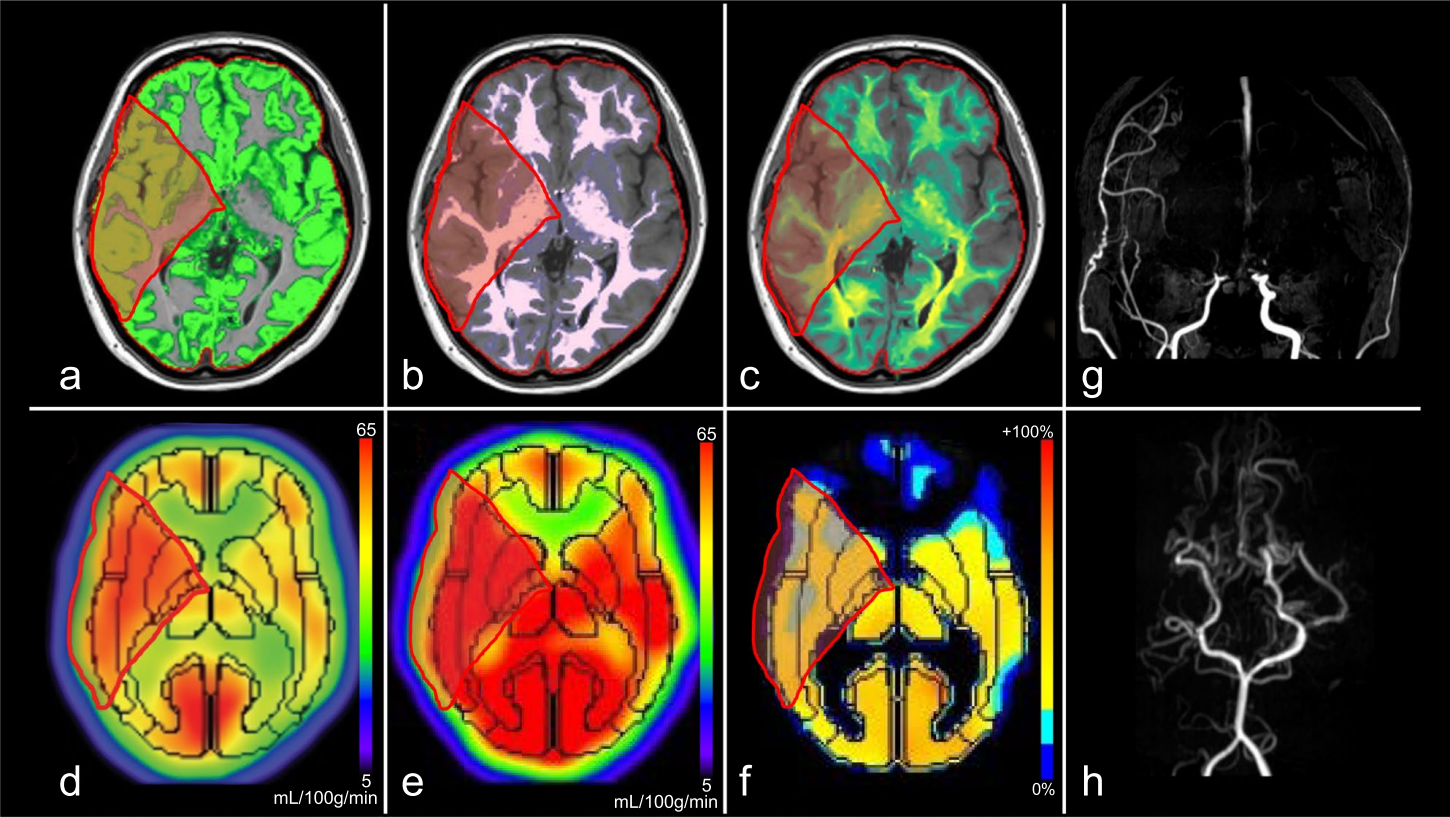
Images from a female patient in her 20 s with moyamoya disease (Case 1, Suzuki stage: right, III; left III, see (**g**,**h**)). (**a**) Gray matter map, (**b**) white matter map, (**c**) myelin-correlated map, (**d**) ^123^I-IMP cerebral blood flow (resting state), (**e**) ^123^I-IMP cerebral blood flow (acetazolamide challenging), (**f**) cerebrovascular reactivity, (**g**) MRA-MIP (anterior circulation), (**h**) MRA-MIP (posterior circulation). Volume fraction maps reveal each fraction. The gray matter is preserved in the right middle cerebral arterial territory (red area: (**a**) 12.6%, (**b**) 5.9%, and (**c**) 1.8%). Both resting and acetazolamide cerebral blood flow maps are maintained in the same territory ((**d**) 0.99 and (**e**) 0.96, corrected by cerebellar hemisphere), and there is a slight decrease in cerebrovascular reactivity ((**f**) –3.5%). ^*123*^*I-IMP*, iodine-123-N-isopropyl-p-iodoamphetamine; *MRA-MIP*, magnetic resonance angiography-maximum intensity projection.

**Figure 3:**
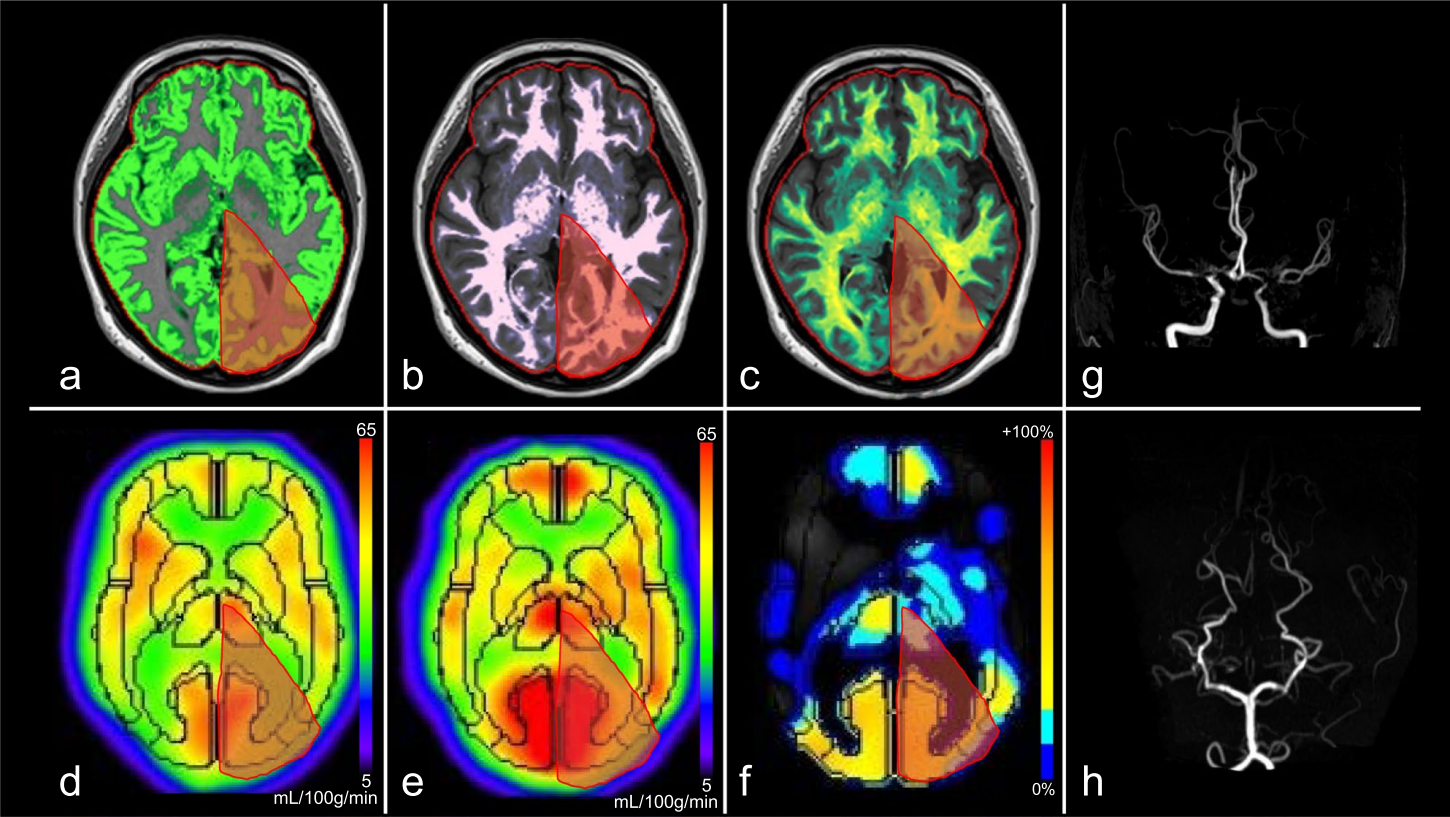
Images from a female patient in her 30 s with moyamoya disease (Case 2, Suzuki stage: right, II; left III, see (**g**,**h**)). (**a**) Gray matter map, (**b**) white matter map, (**c**) myelin-correlated map, (**d**) ^123^I-IMP cerebral blood flow (resting state), (**e**) ^123^I-IMP cerebral blood flow (acetazolamide challenging), (**f**) cerebrovascular reactivity, (**g**) MRA-MIP (anterior circulation), (**h**) MRA-MIP (posterior circulation). Volume fraction maps reveal each fraction. The gray matter is preserved in the left posterior cerebral arterial territory (red area: (**a**) 11.9%, (**b**) 3.9%, and (**c**) 1.5%). Both resting and acetazolamide cerebral blood flow maps are maintained in the same territory ((**d**) 0.99 and (**e**) 1.32, corrected by cerebellar hemisphere), and there is an increase in cerebrovascular reactivity ((**f**) 33.5%). The WAIS test reveals an average score (FSIQ 102). ^*123*^*I-IMP*, iodine-123-N-isopropyl-p-iodoamphetamine; *FSIQ*, Full-Scale Intelligence Quotient; *MRA-MIP*, magnetic resonance angiography-maximum intensity projection; *WAIS*, Wechsler Adult Intelligence Scale.

**Figure 4:**
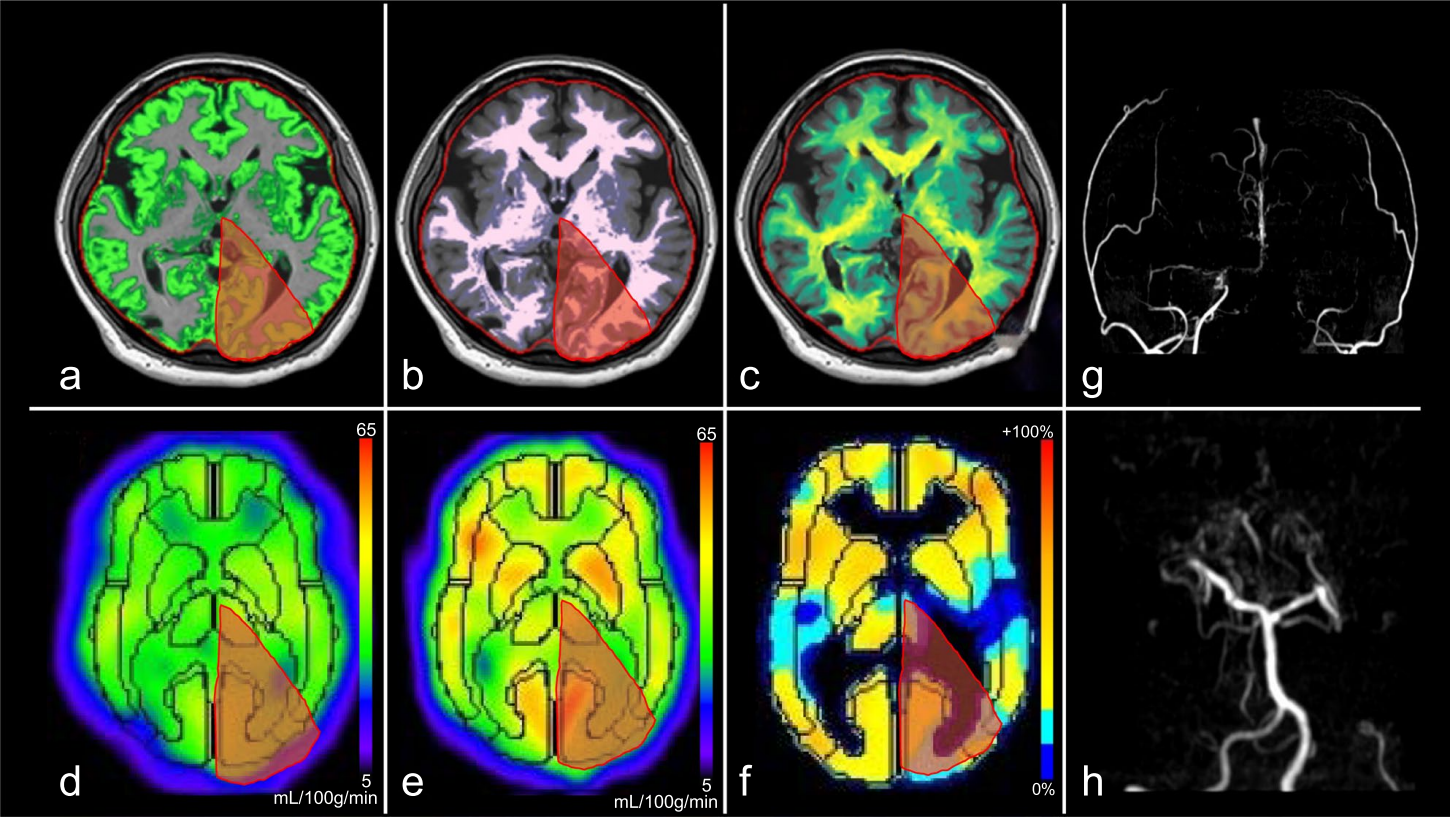
Images from a female patient in her 40 s with moyamoya disease (Case 3, Suzuki stage: right, V; left VI, see (**g**,**h**)). (**a**) Gray matter map, (**b**) white matter map, (**c**) myelin-correlated map, (**d**) ^123^I-IMP cerebral blood flow (resting state), (**e**) ^123^I-IMP cerebral blood flow (acetazolamide challenging), (**f**) cerebrovascular reactivity, (**g**) MRA-MIP (anterior circulation), (**h**) MRA-MIP (posterior circulation). Volume fraction maps reveal each fraction. The gray matter is decreased in the left posterior cerebral arterial territory (red area: (**a**) 6.0%, (**b**) 3.2%, and (**c**) 1.0%). Resting cerebral blood flow is preserved, but the acetazolamide cerebral blood flow map is decreased in the same territory ((**d**) 0.98 and (**e**) 0.98, corrected by cerebellar hemisphere), and there is a moderate decrease in cerebrovascular reactivity ((**f**) –8.9%). The WAIS test reveals a low score (FSIQ 49). ^*123*^*I-IMP*, iodine-123-N-isopropyl-p-iodoamphetamine; *FSIQ*, Full-Scale Intelligence Quotient; *MRA-MIP*, magnetic resonance angiography-maximum intensity projection; *WAIS*, Wechsler Adult Intelligence Scale.

Representative images of patients with moyamoya disease are presented in Figs. 2, 3, 4. Taken together, the results of these cases suggest a positive correlation between brain volume and CBF and a negative correlation between Suzuki stenosis grade and CVR.

### Patient demographics

A total of 18 patients (median age, 44 years; interquartile range, 38.3–50.0 years; three male and 15 female patients) were included in this study. All 18 patients were right-handed. An overview of patient demographics and characteristics, as well as Suzuki staging, is presented in Table 1.

**Table 1:** Patient demographics, Suzuki staging, and brain functional test results.

No	Age [year]	Sex	Suzuki staging	WAIS					WMS-R				
			R, L	FSIQ	VCI	PRI	WMI	PSI	Verbal	Visual	General	Attention	Delayed
1	54	M	3, 4	102	97	109	92	110	96	96	95	107	100
2	72	M	4, 4	98	95	102	85	89	70	93	76	86	101
3	40	M	3, 4	95	91	101	85	110	74	99	78	115	85
4	45	M	5, 6	49	56	50	54	54	65	40	53	50	40
5	39	F	4, 3	71	73	74	81	69	104	78	96	92	98
6	47	M	5, 4	58	79	66	67	50	57	60	52	63	61
7	25	F	3, 3	98	104	103	88	93	99	101	100	87	105
8	39	F	3, 3	103	102	99	109	96	95	106	98	110	108
9	43	M	2, 3	100	90	103	100	108	98	106	100	117	111
10	31	F	2, 3	102	98	103	100	102	113	114	115	93	112
11	41	M	2, 3	73	79	76	82	77	77	111	84	92	84
12	53	M	4, 3	95	88	105	105	97	93	114	99	105	106
13	44	F	3, 2	81	71	95	74	92	104	106	105	94	104
14	49	F	2, 3	97	100	101	114	75	96	94	94	97	103
15	38	F	3, 3	77	81	78	74	63	84	62	75	90	75
16	20	M	4, 3	81	78	88	102	102	98	112	102	110	106
17	45	M	4, 2	96	94	101	94	99	89	104	92	101	96
18	59	M	3, 4	112	117	107	117	93	113	120	117	117	122

*Attention*, Attention/Concentration Index; *Delayed*, Delayed Recall Index; *FSIQ*, Full-Scale Intelligence Quotient; *General*, General Memory Index; *L*, left cerebral hemisphere; *PRI*, Perceptual Reasoning Index; *PSI*, Processing Speed Index; *R*, right cerebral hemisphere; *VCI*, Verbal Comprehension Index; *Verbal*, Verbal Memory Index; *Visual*, Visual Memory Index; *WAIS*, Wechsler Adult Intelligence Scale; *WMI*, Working Memory Index; *WMS-R*, Wechsler Memory Scale-Revised; *yr*, year-old.

### Brain volume versus brain blood flow

**Figure 5:**
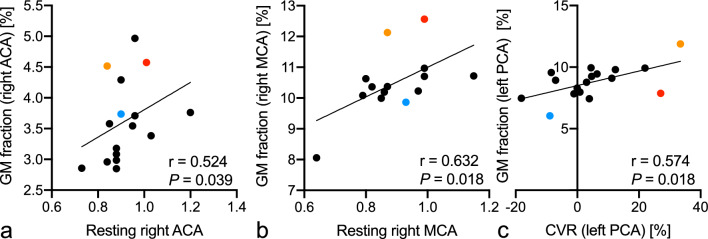
Relationships between brain volume and cerebral blood flow. Significant positive relationships are identified between the resting right anterior cerebral arterial territory and gray matter fraction (a; *r* = 0.524, *P* = 0.039), between the resting right middle cerebral arterial territory and gray matter fraction (b; *r* = 0.632, *P* = 0.018), and between the cerebrovascular reactivity and gray matter fraction (c; *r* = 0.574, *P* = 0.018). The red, orange, and blue points indicate cases 1, 2, and 3 (Figs. 2, 3, 4), respectively. *ACA*, anterior cerebral arterial territory; *CVR*, cerebrovascular reactivity; *GM*, gray matter; *MCA*, middle cerebral arterial territory; *PCA*, posterior cerebral arterial territory.

Regarding the correlations between brain volume and brain blood flow, only three were statistically significant (Fig. 5, Table 2). The resting CBF was positively correlated with the GM fractions in the right ACA and right MCA territories (*r* = 0.524, 0.632; *P* = 0.039, 0.018, respectively). The CVR was positively correlated with the GM fraction in the right PCA territory (*r* = 0.574; *P* = 0.018).

**Table 2:** Volume fractions for each arterial territory.

No	Volume fraction [%]																	
	R									L								
	ACA			MCA			PCA			ACA			MCA			PCA		
	GM	WM	MyC	GM	WM	MyC	GM	WM	MyC	GM	WM	MyC	GM	WM	MyC	GM	WM	MyC
1	3.2	1.5	0.6	8.4	6.0	2.2	6.9	4.9	1.7	3.3	1.5	0.6	11.1	7.1	2.9	9.9	5.0	2.0
2	3.4	1.8	0.7	10.7	7.2	2.5	7.7	4.4	1.4	3.7	1.6	0.6	10.6	6.0	2.1	8.3	3.8	1.3
3	2.9	1.7	0.7	10.6	8.9	3.3	7.9	5.2	1.9	3.0	1.9	0.7	12.0	9.4	3.4	8.0	4.5	1.6
4	3.7	1.4	0.5	9.9	6.7	2.3	5.5	3.3	1.0	3.3	1.4	0.5	12.1	7.1	2.4	6.0	3.2	1.0
5	4.5	1.9	0.6	8.1	5.8	1.9	7.5	4.1	1.5	4.1	2.3	0.8	9.1	7.5	2.6	7.8	5.2	1.7
6	3.2	1.4	0.5	9.7	7.3	2.3	10.7	5.7	1.8	3.4	1.8	0.6	9.8	8.0	2.7	6.9	2.5	0.8
7	3.6	1.3	0.5	10.1	5.3	2.1	9.5	3.6	1.2	3.6	1.4	0.6	9.9	5.2	2.0	9.8	3.7	1.3
8	3.0	1.3	0.5	10.0	5.5	2.0	8.3	3.5	1.2	2.9	1.2	0.5	10.5	5.6	2.1	9.6	3.5	1.2
9	5.0	1.9	0.6	11.0	6.6	2.2	8.5	3.8	1.3	3.2	2.2	0.7	10.2	6.7	2.3	8.8	4.5	1.6
10	4.3	1.5	0.7	12.1	4.9	1.9	9.5	3.3	1.3	3.8	1.6	0.7	12.0	4.9	1.8	11.9	3.9	1.5
11	3.7	1.3	0.5	9.9	6.1	2.1	8.2	3.6	1.3	3.4	1.6	0.6	12.9	6.0	2.3	7.4	4.1	1.6
12	3.8	1.5	0.6	10.7	7.0	2.6	7.8	4.3	1.5	3.8	1.7	0.6	11.4	7.3	2.7	7.5	3.7	1.3
13	3.1	1.3	0.5	10.2	6.0	2.2	9.8	3.5	1.4	2.6	1.2	0.5	10.4	6.1	2.2	9.1	3.9	1.4
14	2.8	1.2	0.5	10.4	6.6	2.4	9.1	3.9	1.3	2.7	1.0	0.5	10.4	5.8	2.1	8.9	4.0	1.3
15	2.7	1.2	0.6	11.0	5.1	1.8	9.5	3.8	1.3	3.0	1.0	0.5	10.6	5.7	2.1	9.9	3.6	1.2
16	4.6	1.7	0.5	12.6	5.9	1.8	8.0	3.2	1.0	4.4	1.9	0.7	12.3	6.2	2.0	7.9	3.2	1.1
17	3.0	1.1	0.5	10.4	5.4	2.1	9.3	4.0	1.5	3.1	1.1	0.4	10.8	5.8	2.2	9.4	3.6	1.3
18	3.5	1.5	0.6	10.2	6.7	2.8	8.3	3.8	1.6	3.4	1.3	0.6	11.7	7.3	2.9	9.2	4.5	1.7

*ACA*, anterior cerebral arterial territory; *GM*, gray matter volume; *L*, left hemisphere; *MCA*, middle cerebral arterial territory; *MyC*, myelin-correlated; *PCA*, posterior cerebral arterial territory; *R*, right hemisphere; *WM*, white matter.

### Brain volume versus brain function

With regard to the correlations between brain volume and brain function, only three showed significance. The FSIQ and VCI scores were positively correlated with the GM fractions in the left PCA territory (*r* = 0.625, 0.615; *P* = 0.009, 0.010, respectively). The PSI score was positively correlated with the GM fraction in the right MCA territory (*r* = 0.665; *P* = 0.011). However, no correlations were observed between brain volume and brain function after Bonferroni correction (corrected *P*-value = 0.0027). There were no correlations between brain volume and brain memory indices assessed by WAIS-R.

### Suzuki’s stenosis grade vs. brain volume and brain blood flow

A negative correlation was observed between Suzuki’s stenosis grade and the CVR (*P* = 0.0044).

## Discussion

In the current study, we clarified the relationships between brain volume and brain blood flow and between brain volume and brain function in patients with moyamoya disease using SyMRI volumetry. Representative cases showed a positive correlation between brain volume and CBF and a negative correlation between Suzuki stenosis grade and CVR. With respect to CBF, the right anterior circulation was positively correlated with the GM fraction. Regarding CVR, only the right posterior circulation was positively correlated with the GM fraction. In terms of brain function, the FSIQ and VCI scores showed marginally positive correlations with GM fractions in the left PCA territory, and the PSI score showed a marginally positive correlation with the GM fraction in the right MCA territory. In addition, Suzuki’s stenosis grade was negatively correlated with the CVR.

The GM fraction was positively correlated with CBF but not with WM or MyC in our study. Hara et al. reported correlations between WM and CBF and between myelin and CBF^18,19^. This difference may be due to the different study subjects; the previous report found differences in WM and myelin between healthy individuals and patients with moyamoya disease, but in the present study, which included only the patient group, these differences may not have been apparent or appeared as GM differences. Some reports have shown that the cerebral cortex is vulnerable to ischemia^20,21^; indeed, reduced GM volume has been reported in patients with moyamoya disease^22,23^. Progressive stenosis at the terminal of internal carotid arteries causes severe ischemia in the anterior circulation in moyamoya disease. The CVR correlated with the GM fraction in the left PCA territory. In moyamoya disease, the development of leptomeningeal anastomoses in the posterior circulation compensates for ischemia in the anterior circulation^24^, thus preserving CVR in the posterior circulation, which may be why the GM fraction was also preserved. Suzuki’s stenosis grade correlated only with CVR, but not with brain volume or CBF. This is because in patients with moyamoya disease, imaging stenosis does not correlate linearly with reduced CBF due to the compensatory development of collateral vessels, hence the need for acetazolamide loading, which is consistent with previous findings^25^. Our study demonstrated that brain volume measured by SyMRI could predict the CBF in that territory. This study found that CBF positively correlated with GM volume, maintaining CBF is important for neurons, and revascularization therapy may be important for neuron protection.

We found marginally positive correlations between FSIQ/VCI scores and GM fraction in the left PCA territory. This region of the brain is involved in language processing and verbal abilities, which are both important components of overall intelligence. Previous studies have shown that damage to the left PCA territory can lead to language impairments, such as aphasia, suggesting that this region is critical for language function^26,27^. Hosoda et al. reported that FSIQ/VCI correlated with oxygen metabolism in the occipital lobe (PCA territory)^28^, which is consistent with the results of this study. We also revealed that the PSI score was positively correlated with the GM fraction in the right MCA territory. The right cerebral hemisphere is particularly important for spatial and non-verbal abilities^29^. Therefore, damage to or reduction in GM volume in this region may lead to impaired cognitive functions such as attention, memory, and processing speed, which could manifest as a decrease in PSI. Hara et al. reported that PSI correlated with intra-neurite volume fraction in the frontoparietal lobes (MCA territory)^30^. Nakamizo et al. reported that the right MRA total and right PCA scores were negatively associated with 5-year changes in the total frontal assessment battery score and total neurobehavioral cognitive status examination score^31^. These results are consistent with those of the present study. We demonstrated that brain function tended to be maintained in territories where brain volume is preserved.

Our study had some limitations. First, the study population was small. To compensate for interindividual variability, we used cerebellar-corrected rCBF, which is not the absolute value. We used a mixture of WAIS-III/IV intelligence tests, but as these are interchangeable, we determined that this would not majorly affect our results. The results of this study are preliminary and further studies with larger sample sizes are necessary to confirm our results.

In conclusion, territorial GM fraction measured by SyMRI correlates with CBF and brain function in adult moyamoya disease. SyMRI can quantitatively assess brain volume loss in patients with moyamoya disease and may help determine disease severity and strategies for revascularization therapy.

## Data Availability

The datasets used and/or analyzed during the current study are available from the corresponding author on reasonable request.
